# CCN1 is a therapeutic target upregulated in EML4-ALK mutant lung adenocarcinoma reversibly resistant to alectinib

**DOI:** 10.1038/s41419-025-07601-4

**Published:** 2025-04-15

**Authors:** Yihua Huang, Jie Huang, Jianhua Zhan, Maojian Chen, Jiani Zheng, Junyi He, Wenfeng Fang, Li Zhang, Jing Li

**Affiliations:** 1 Department of Medical Oncology, State Key Laboratory of Oncology in South China, Guangdong Provincial Clinical Research Center for Cancer, Sun Yat-sen University Cancer Center, Sun Yat-sen University, Guangzhou, China; 2 Phase I Clinical Trial Centre, Sun Yat-sen Memorial Hospital, Sun Yat-sen University, Guangzhou, China; 3 Guangdong Provincial Key Laboratory of Malignant Tumor Epigenetics and Gene Regulation, Guangdong-Hong Kong Joint Laboratory for RNA Medicine, Sun Yat-Sen Memorial Hospital, Sun Yat-Sen University, Guangzhou, China; 4 Department of Medical Oncology, Xiamen Key Laboratory of Antitumor Drug Transformation Research, the First Affiliated Hospital of Xiamen University, School of Medicine, Xiamen University, Xiamen, China

**Keywords:** Cancer therapy, Lung cancer

## Abstract

There is limited understanding of the phenomenon of reversible drug resistance, which is characterized by tumor cells regaining sensitivity when the drug is changed or withdrawn after a period of drug resistance. This phenomenon is usually not associated with genetic alterations of tumor cells. In this study, reversible resistant state was induced by alectinib in EML4-ALK mutant lung cancer cell. By performing RNA sequencing on reversible drug-resistant cell line to examine changes in transcriptional profile, significant change in CCN1 was detected after withdrawal and repeated administration of alectinib. Targeting CCN1 resulted in inhibition of tumor cell proliferation and angiogenesis, and restoration of sensitivity to alectinib in reversible drug-resistant cells. Further studies revealed that CCN1 could affect the expression of VEGFA by affecting AKT phosphorylation, and the change of NF-κB could impact the activation of CCN1-AKT-VEGFA pathway. Suppressing NF-κB or CCN1 receptor could improve the sensitivity to alectinib, further suggesting that NF-κB and CCN1 might play a key role in overcoming reversible drug resistance.

## Introduction

Lung cancer is the leading cause of cancer mortality worldwide [1]. Non-small cell lung cancer (NSCLC) accounts for ~85% of lung cancers and exhibits a remarkable frequency of oncogenic driver alterations. Over the past two decades, the discovery of oncogenic driver alterations and the development of matched molecularly targeted therapies have contributed to a substantial reduction in NSCLC mortality [2, 3]. A variety of ALK gene alterations, including ALK fusion, point mutations, and amplification, have been identified across various tumor types. The oncogenic ALK can be activated by ALK fusions in several cancers, such as NSCLC, anaplastic large cell lymphoma (ALCL), as well as by point mutations in neuroblastoma and anaplastic thyroid cancer [4, 5]. Additionally, ALK amplification as a negative prognostic factor has also been reported in certain cancers [6, 7].

Chromosomal rearrangements involving ALK have been identified as an oncogenic driver in 3–7% of patients with NSCLC and have been associated with non-smoking status, younger age, and adenocarcinoma histology [8]. Although the development of ALK tyrosine kinase inhibitors (TKIs) has revolutionized the treatment landscape for ALK-driven NSCLC patients, acquired resistance is inevitable and remains a critical unmet challenge [9, 10].

Much effort has been devoted to elucidating the mechanisms of acquired resistance in order to develop subsequent therapies. The resistance mechanisms of ALK TKIs can be categorized as either “on-target” such as ALK aberrations, or “off-target” involving the activation of bypass pathways, downstream pathways, or phenotypic transformation [10, 11]. Despite the improved understanding of resistance mechanisms, little is known about the changes that occur during the evolution of acquired resistance. Previous studies have reported the “reversible resistance phenomenon” in the clinical setting, which manifested by the fact that some patients resistant to targeted agents could regain sensitivity after a period of treatment discontinuation [12–15]. The “reversible resistance phenomenon”, which could be reverted, may indicate an incompletely resistant state resulting from non-genomic mechanisms. The study of reversible resistance would undoubtedly promote a better understanding of resistance transformation and optimize clinical medication.

In this study, we established an ALK-mutant lung cancer cell line that was unstably resistant to alectinib, the second-generation ALK inhibitor. Genomic sequencing did not identify known resistant mutations. Therefore, RNA sequencing was performed to investigate potential changes in the expression landscape. We found that CCN1, which encoded a secreted matricellular protein, showed obvious changes when alectinib was withdrawn or rechallenged. Further experiments revealed that CCN1 was required for growth and angiogenesis ability via AKT-VEGFA signaling in unstable resistant cells. Targeting CCN1 could reverse resistance to alectinib. Our study provides new insights into the therapeutic targets or resistance biomarkers of ALK mutant NSCLC.

## Materials and methods

### Cell culture and reversible drug resistance cell line construction

The human lung cancer cell line H3122 was obtained from the American Type Culture Collection (ATCC). Short tandem repeat (STR) analysis was performed to authenticate the cells, and routine screening for mycoplasma contamination was conducted. All cells were cultured under standard conditions using RPMI 1640 medium supplemented with 10% fetal bovine serum (FBS) and 1% penicillin-streptomycin (P/S).

To induce reversible resistance, H3122 cells were continuously exposed to sequentially increasing concentrations of alectinib. After reaching a concentration of 2 μM, the cells were maintained in culture medium containing 2 μM alectinib for 3 months. The reversible drug resistance of the alectinib-treated cell line was confirmed by assessing drug sensitivity both during the alectinib maintenance period and 2 weeks after alectinib withdrawal.

### Drug holiday-administration-holiday experimental cycles

In order to clarify the mechanism of reversible drug resistance, we performed a cycle of drug holiday-administration-holiday experiment. Briefly, the reversibly resistant cell lines were pre-maintained at 2 μM alectinib, and cells were collected at baseline. The period duration of drug withdrawal, rechallenge, and re-withdrawal was each 2 weeks, and cells were collected on day 3, day 7, and day 14 of each period, respectively. Cell samples harvested during the process were used for subsequent RNA sequencing.

### Cell transfection

Lentiviral expression plasmids were synthesized from Tsingke Biotechnology (Beijing, China). These included the pLVX-CMV-PURO vectors subcloned with CCN1. The CCN1 plasmid was transfected into HEK293T cell line together with the packaging and envelope plasmids (psPAX2, pMD2.G) using Lipofectamine™ 3000 Reagent (#L3000008, Invitrogen). Lentivirus-containing cell culture supernatant was then collected, filtered through a 0.45 μm syringe, and immediately added to the target cells along with polybrene. Pooled target cell populations were then selected with 2 μg/ml of puromycin. CCN1 siRNA was synthesized from RIBO Biotechnology (Guangzhou, China) and transfected into cells using Lipofectamine™ RNAiMAX Reagent (#13778075, Invitrogen). The sequence of siCCN1 is CCAGAAATGTATTGTTCAA.

### Whole-genome sequencing and RNA sequencing

Whole-genome sequencing (WGS) was performed in H3122 and H3122 alectinib resistant (AR) cells to identify gene alterations that emerged following long-term treatment with alectinib. Transcriptome changes during holiday-administration-holiday experiments were confirmed by RNA sequencing. Genomic DNA and total RNA were extracted using the QIAamp DNA Mini Kit (#51306, Qiagen) and MiniBEST Universal RNA Extraction Kit (#9767, TaKaRa) according to the manufacturer’s instructions, respectively. WGS libraries were constructed using MGIEasy Fast FS Library Prep Set (#940-001197-00, MGI Tech, China). RNA-seq libraries were constructed using MGIEasy Fast RNA Library Prep Set (#940-000890-00, MGI Tech, China). WGS and RNA sequencing procedures were performed on a DNBSEQ-T7RS sequencer (MGI Tech, China) with 150-bp paired-end reads according to the manufacturer’s recommendations. The raw sequence data reported in this paper have been deposited in the Genome Sequence Archive [16] in National Genomics Data Center [17], China National Center for Bioinformation/Beijing Institute of Genomics, Chinese Academy of Sciences (GSA-Human: HAR009333), which are publicly accessible at https://ngdc.cncb/ac.cn/gsa-human.

### Sequencing data analysis

The WGS sequenced reads were mapped to the reference genome (GRCh37) using BWA [18] after removing adaptor and low-quality reads using fastp [19]. Duplicated reads were identified by unique identifiers and were ignored in subsequent analysis. Single-nucleotide variants (SNVs) and indels were identified using MuTect2 [20] and annotated using VEP [21]. Structural variation (SV) was identified using Manta [22], and copy number variations (CNVs) were identified using GATK [23]. For RNA sequencing, the expression of the transcripts was quantified against the reference genome (GRCh37) using Salmon [24]. maSigPro [25, 26] was used to analyze the time-course expression data to find genes most associated with drug treatment.

### Real-time quantitative PCR (qPCR)

Total RNA was extracted from tumor cells using MiniBEST Universal RNA Extraction Kit (#9767, TAKARA) and reverse transcribed using PrimeScript™ RT reagent Kit with gDNA Eraser (#RR047A, TAKARA). TB Green™ Premix Ex Taq™ (Tli RNaseH Plus) (#RR820A, TAKARA) was used as a fluorescent DNA binding dye, and RT-PCR was performed on a Biorad system (Applied Biosystems, California, USA). Gene expression was normalized to that of GAPDH. The following primers were used to amplify CCN1 [5′-AATGGAGCCTCGCATCCTATA-3′(forward) and 5′- TTCTTTCACAAGGCGGCA-3′(reverse)], CCN2 [5′-CCCTCGCGGCTTACCGACTGG-3′(forward) and 5′-CACAGGTCTTGGAACAGGCGC-3′(reverse)] and GAPDH [5′- GTCTCCTCTGACTTCAACAGCG-3′(forward) and 5′-ACCACCCTGTTGCTGTAGCCAA-3′ (reverse)].

### Cell viability and colony formation assays

Cell viability was assessed using the Cell Counting Kit-8 kit (#C0005, Dojindo). Cells were collected from the suspension medium, spun down at 300 g for 5 min, and resuspended in fresh RPMI medium. 1000 cells per well were plated in 96-well plates. CCK-8 solution (10 μl) mixed with medium was added to each well on a given day. After incubation at 37 °C for 2 h, the absorbance was measured at 450 nm. For the drug sensitivity analysis, 5000 cells per well were plated in 96-well plates. Cells were treated with ten different concentrations of inhibitors in serial dilutions or vehicle alone to a final volume of 100 μl per well. After 72 h, Cell Counting Kit-8 was added to each well. The half-maximal inhibitory concentration (IC50) values were calculated using GraphPad Prism 10 at 50% inhibition. For colony formation assays, 1000 cells were seeded in 6-well plates. After 24 h, 100 nM of alectinib was added to maintain the resistant state of H3122 AR cells in the whole process. Cell colonies were fixed with methanol and stained with 0.3% crystal violet before being photographed. Each experiment was replicated three separate times.

### Western blotting

Total protein was extracted using RIPA lysis buffer with protease inhibitor cocktail. Proteins from each group were loaded onto SDS-PAGE gels and separated before transfer to PVDF membranes. After blocking with skim milk for 1 h at room temperature, the membrane was incubated with primary antibodies against each target protein overnight at 4 °C. The membrane was then incubated with secondary antibodies for 1 h at room temperature. Primary antibodies included ALK (#3633), pALK (#3341), pAKT (#9271), AKT (#4691), pERK (#4370), ERK (#4695), STAT3 (#4904), pSTAT3 (#9143), CCN1 (#14479), CCN2 (#10095), FAK (#3285), pFAK (#8556), NF-κB (#8242), pNF-κB (#3033), VEGFR2 (#2479), YAP (#14074), TGF-β (#3711) from Cell Signaling Technology and VEGFA (#ab46154) from Abcam. Blots were probed with antibodies against GAPDH (Proteintech, #10494-1-AP) as a loading control.

### Scratch wound healing assays

Cells with different treatments were cultured in six-well plates at 37 °C. Scratch wounds were made by using the fine end of 10 μl pipette tips. Images of migrated cells were taken at different times and analyzed using the Image J software.

### Tube formation assay

HUVECs were cultured for tube formation assay. Supernatants from tumor cells with different treatments were collected and stored at −80 °C. The µ-Slide plates (#81506, Ibidi) were coated with Matrigel (#354234, Corning) and incubated at 37 °C for 30 min to allow gelation. HUVECs were diluted with various supernatants and plated at a density of 10,000 cells per well. Cells were incubated at 37 °C with 5% CO_2_, and images were captured within 6 h. The tubular structures were photographed under light microscope and analyzed using the Image J software.

### Statistical analysis

GraphPad Prism 10 was used to generate graphs and statistics. Unpaired Student’s *t*-test was used to analyze the differences between the two groups. The results are presented as means ± standard. Each experiment was replicated three separate times. Statistical significance was defined as a two-sided *P* value < 0.05.

## Results

### High-concentration of alectinib induced reversible drug resistance in EML4-ALK mutant lung adenocarcinoma cells

**Fig. 1:**
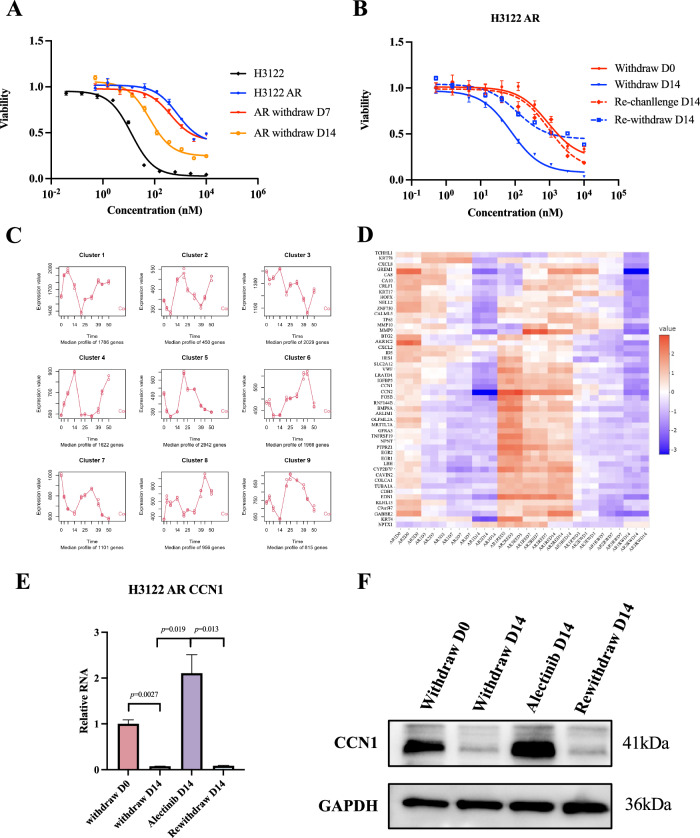
CCN1 changed as ALK inhibitor treatment altered. **A** Alectinib sensitivity detection in H3122 cells, H3122 AR cells, and AR cells after alectinib withdrawal. **B** Alectinib sensitivity detection in H3122 AR before and after alectinib withdrawal, and alectinib rechallenge. **C** MaSigPro analysis for samples collected at different times during alectinib holiday-administration-holiday period. **D** Top 50 genes in clusters 5, 7, and 9 with similar RNA changes. **E** CCN1 RNA level detection at different times during alectinib holiday-administration-holiday period. **F** CCN1 protein level detection at different times during alectinib holiday-administration-holiday period. Error bars reflect mean ± standard deviation.

To construct adenocarcinoma cells resistant to alectinib, high concentration of alectinib was used to incubate human H3122 cells carrying EML4-ALK fusion. The concentration of alectinib was gradually increased and maintained at 2 µM when the cancer cells could survive and grow with TKI treatment. After the cells could well tolerate with 2 µM of alectinib, the mean IC50 of alectinib in alectinib-treated H3122 cells (H3122 AR) reached 614.1 ± 154.7 nM, over 10-fold higher than that of the parental H3122 cells (10.71 ± 3.869 nM) (Fig. 1A). WGS was performed in H3122 and H3122 AR cells to identify gene alterations following long-term treatment with alectinib and investigate potential genomic-resistance mechanisms. Although a great number of genetic mutations were identified, most alterations did not change the amino acid sequence (Tables S1 and S2; Fig. S1A). Only 62 nonsynonymous mutations were detected and did not include any ALK-dependent mutations leading to resistance or any off-target mechanisms such as HER2 amplification (Table S3 and Fig. S1B). Alectinib was withdrawn to determine whether the reversible resistant phenotype was constant. The IC50 decreased after 1 week of drug withdrawal (326.0 ± 85.84 nM) and became lower after 2 weeks (80.28 ± 23.07 nM) (Fig. 1A). The improved sensitivity after alectinib withdrawal suggested that resistance to alectinib was unstable. Therefore, we confirmed that the resistant state of H3122 AR was reversible and did not result from genetic aberrations.

### RNA sequencing found that CCN1 changed as ALK inhibitor treatment altered

To confirm the observation of the reversible resistance to alectinib, we performed drug holiday-administration-holiday experimental cycles to the H3122 AR cells. As shown in Fig. 1B, after 2 weeks of drug withdrawal, increased sensitivity to alectinib was observed (IC50 fell from 940.4 ± 168.9 nM to 68.80 ± 13.36 nM). Then IC50 returned to 861.9 ± 196.3 nM after 2 weeks of alectinib rechallenge and decreased to120.6 ± 10.71 nM after repeated alectinib withdrawal (Fig. 1B). Therefore, the reversible resistance phenomenon was determined in response to TKI treatment. Given that such resistance is not associated with genetic alterations, non-genetic regulation might play an important role during the process. Thus, RNA samples were collected across the drug holiday-administration-holiday experiment on day 0, day 3, day 7, and day 14 for transcriptome analysis. We hypothesized that there should be differences in the transcriptome of the cells during the drug holiday-administration-holiday experimental cycles, with these differences remaining consistent over time. According to the gene expression profiles throughout the time series, we employed MaSigPro to categorize the genes into clusters, ultimately identifying 9 distinct clusters exhibiting varied temporal patterns. Further analysis of these clusters led to the identification of three gene clusters (as shown in Fig. 1C, clusters 5, 7, and 9) that were most likely implicated in drug resistance. These clusters demonstrated a tendency to be down-regulated during drug interruption and up-regulated upon drug rechallenge, suggesting a possible direct role in mediating drug resistance. Given the similar temporal profiles of these three clusters, we consolidated them to investigate the genes that might be important on this reversibly resistant phenomenon. Among the top 50 genes in these 3 clusters, CXCL family, such as CXCL8, CXCL2, and matrix metalloproteinases (MMPs), such as MMP9, MMP10, emerged. Two members of the cellular communication network (CCN) family, CCN1 and CCN2, were noticed (Fig. 1D). Previous studies have reported that CCN1 and CCN2 are associated with tumor growth, migration, and drug resistance [27–29]. However, the role of CCN family in lung cancer remains controversial. qPCR confirmed the expression changes of CCN1 and CCN2 (Figs. 1E and S2A), agreeing with the transcriptomic data. CCN1 protein level also mirrored the changes on RNA level (Fig. 1F). However, CCN2 protein did not change accordingly (Fig. S2B). Taken together, we found that CCN1 was an important factor that tended to be downregulated after drug withdrawal, upregulated after drug rechallenge, and downregulated again after drug withdrawal in both RNA and protein levels.

### Tumor cell proliferation and angiogenesis can be inhibited and drug sensitivity to alectinib can be enhanced by inhibiting CCN1 in H3122 AR cells

**Fig. 2:**
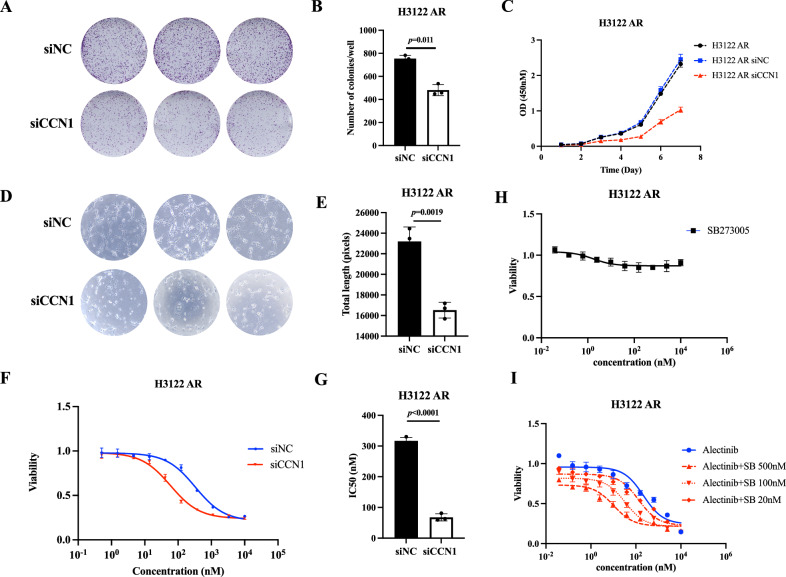
CCN1 could affect the ability of growth and angiogenesis, and alectinib sensitivity in H3122 AR cells. **A** Colony forming experiment of H3122 AR cells after CCN1 knockdown. **B** CCN1 knockdown significantly decreased the colony forming capacity of H3122 AR cells. **C** The growth curve of H3122 AR cells after CCN1 knockdown. **D** Tube formation assay of H3122 AR cells after CCN1 knockdown. **E** CCN1 knockdown significantly decreased the angiogenesis capacity of H3122 AR cells. **F**, **G** CCN1 knockdown could improve the alectinib sensitivity in H3122 AR cells. **H** High concentration (10 μM) of SB273005 did not obviously affect the survival of H3122 AR cells. **I** The addition of SB273005 could improve the sensitivity of alectinib in H3122 AR cells. Error bars reflect mean ± standard deviation.

Considering that the resistant state was rapidly changed without alectinib induction, genetic knockdown of CCN1 by siRNA was applied to investigate CCN1 function. By knockdown of CCN1, the colony forming capacity fell by 39% in H3122 AR cells (*p* = 0.0011, Fig. 2A, B). Significant inhibition of cell growth viability was also observed (Fig. 2C). Tube formation assay showed that CCN1 knockdown decreased the vascular length of H3122 AR cells (*p* = 0.0019, Fig. 2D, E). However, there was no significant difference in migration ability between siCCN1 H3122 AR cells and control cells (*p* = 0.6535, Fig. S3). In addition, we found that knockdown of CCN1 could improve the sensitivity to alectinib in H3122 AR cells (median IC50, 310.7 ± 11.32 nM vs 63.22 ± 11.66 nM, *p* < 0.0001, Fig. 2F, G). CCN1 mainly acts by binding to the integrin receptor [29, 30]. Therefore, SB273005, an integrin-targeting agent, was used for combination therapy. As shown in Fig. 2H, high concentration of SB273005 did not obviously affect the survival of H3122 AR cells. However, when combined with SB273005, H3122 AR cells became more sensitive to alectinib, indicating the synergistic effect of the combination regimen (Fig. 2I). In conclusion, genetic knockdown of CCN1 could affect the ability of growth and angiogenesis in H3122 AR cells. Improved sensitivity was demonstrated by targeting CCN1.

### CCN1 affects tumor cells through the AKT-VEGFA pathway

**Fig. 3:**
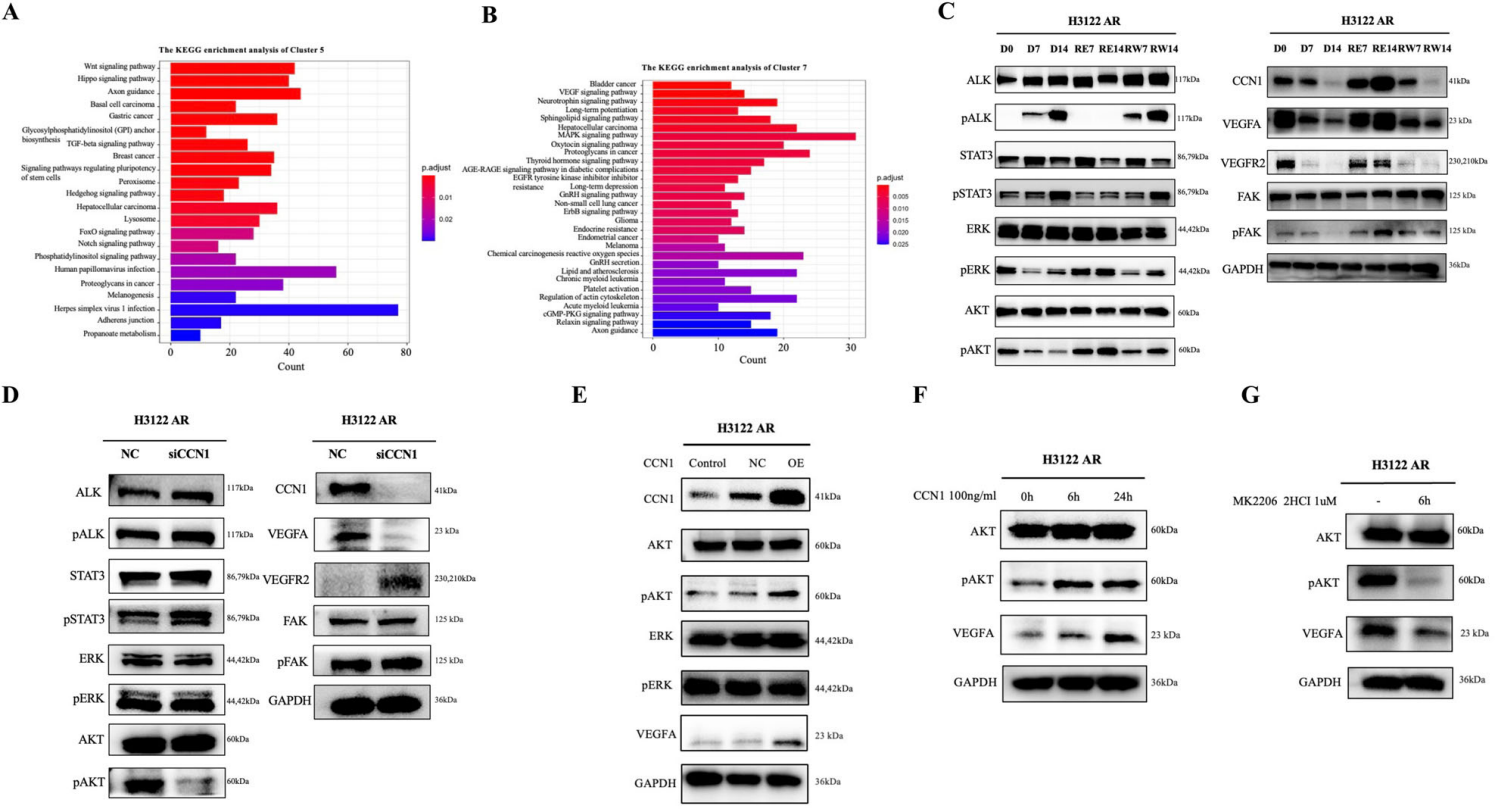
CCN1 affects tumor cells through the AKT-VEGFA pathway. **A**, **B** KEGG analysis for samples collected at different time during alectinib holiday-administration-holiday period (cluster 5, cluster 7). **C** Western blotting of samples collected at different time during alectinib holiday-administration-holiday period. **D** Western blotting of H3122 AR cells after CCN1 knockdown. **E** Western blotting of H3122 AR transfected with CCN1 plasmid. **F** Western blotting of H3122 AR treated with exogenous CCN1. **G** Western blotting of H3122 AR treated with MK2206 2HCI, an AKT inhibitor.

CCN1 could affect the sensitivity to alectinib, growth, and angiogenesis activity of resistant H3122 cells. The underlying mechanism was not clear. KEGG pathway analysis was performed on maSigPro clusters with a similar tendency. Several pathways, such as VEGF pathway, Hippo pathway, and adherens junction pathway, were identified (Figs. 3A, B and S4). Previous studies have shown that CCN1 could promote angiogenesis by activating VEGFA and VEGFR2 [31, 32]. In addition, CCN1 could activate FAK to facilitate cell adhesion and migration [33, 34]. Combined with previous studies, we detected potential proteins that could be associated with CCN1. Several proteins were detected during alectinib withdrawal, rechallenge, and re-withdrawal (Fig. 3C). Consistent with RNA sequencing, CCN1 tended to be downregulated after drug withdrawal and upregulated after drug rechallenge. Abnormality of ALK can activate the downstream RAS-MAPK, PI3K-AKT, and JAK-STAT3 signaling pathways through autophosphorylation. When treated with alectinib, phosphorylation of ALK and STAT3 was inhibited, then recovered after drug discontinuation. However, other downstream proteins, AKT phosphorylation, and ERK phosphorylation had a similar trend with CCN1. VEGFA and VEGFR2, involved in angiogenesis, and FAK phosphorylation, involved in cell adhesion, also changed with CCN1. Further work was done to investigate the underlying regulatory relationships.

CCN1 knockdown was performed to investigate potential regulatory mechanisms in H3122 AR cells. To exclude the influence of ALK, related proteins were also examined. Decreased CCN1 caused obvious decrease in AKT phosphorylation, while no significant changes were observed in the levels of ALK phosphorylation, STAT3 phosphorylation, ERK phosphorylation, suggesting that AKT phosphorylation could be mediated by CCN1 independent of ALK (Fig. 3D). As for the VEGF pathway, a prominent decrease in VEGFA was seen, while VEGFR2 became higher. There was no change in the phosphorylation level of FAK. Similarly, pharmaceutical inhibition of CCN1 by SB273005 demonstrated a significant decrease in the phosphorylation of AKT and VEGFA (Fig. S5). Our results indicated that CCN1 could regulate the activation of AKT phosphorylation independent of ALK. Combining the suppressed angiogenesis activity with the decreased VEGFA level after CCN1 knockdown, we hypothesized that CCN1 might affect blood vessel formation by regulating VEGFA. Overexpression of CCN1 in H3122 AR also induced the expression of phosphorylation AKT and VEGFA, supporting the regulatory function (Fig. 3E). However, overexpression of CCN1 did not confer resistance to alectinib (IC50, NC 189.9 ± 13.51 nM vs CCN1 OE 184.1 ± 22.52 nM, *p* = 0.7215, Fig. S6), suggesting a complicated resistance mechanism in H3122 AR. CCN1 mainly functions as a secreted protein. By exogenous CCN1, increased phosphorylation of AKT and VEGFA was observed in H3122 AR cells (Fig. 3F). On the contrary, exogenous CCN1 did not affect the expression of phosphorylation AKT and VEGFA (Fig. S7) in the parental H3122 cell line, suggesting that H3122 AR might be more susceptible to CCN1 treatment. To explore the regulatory relationships between AKT and VEGFA, MK2206 2HCI, an AKT inhibitor, was used to suppress the phosphorylation AKT, which caused a significant decrease in VEGFA, indicating that VEGFA could be regulated by AKT expression (Fig. 3G). Taken together, we found that in H3122 AR, CCN1 could affect the expression of VEGFA by affecting the phosphorylation level of AKT. Although knockdown of CCN1 could improve the sensitivity to alectinib, overexpression of CCN1 did not cause alectinib resistance, suggesting that the underlying resistance mechanism is complex and CCN1 could serve as a potential therapeutic target.

### CCN1 was activated by NF-κB in unstably resistant cells

**Fig. 4:**
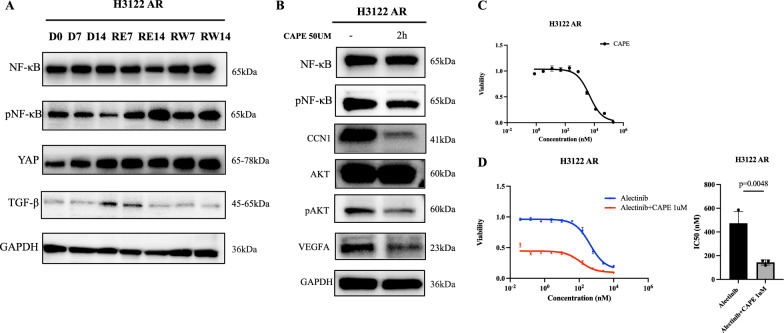
CCN1 was activated by NF-κB in H3122 AR cells. **A** Western blotting of samples collected at different time during alectinib holiday-administration-holiday period. **B** Western blotting of H3122 AR treated with Caffeic Acid Phenethyl Ester (CAPE), an NF-κB inhibitor. **C** CAPE sensitivity in H3122 AR cells. **D** The addition of CAPE could improve the sensitivity of alectinib in H3122 AR cells. Error bars reflect mean ± standard deviation.

To identify the potential upstream factor that could cause CCN1 activation, we detected a number of proteins and found that NF-κB phosphorylation shows the same trend as CCN1 during alectinib holiday-administration-holiday period (Fig. 4A). We further used Caffeic Acid Phenethyl Ester (CAPE), an NF-κB inhibitor, to treat H3122 AR. As shown in Fig. 4B, suppression of NF-κB phosphorylation resulted in decreased CCN1, AKT phosphorylation and VEGFA, supporting that NF-κB could act as an upstream factor (Fig. 4B). Furthermore, CAPE reached a mean IC50 of 5.902 ± 1.302 μM, and the addition of CAPE could improve the sensitivity to alectinib in H3122 AR cells (472.8 ± 98.66 nM vs 142.7 ± 21.45 nM, *p* = 0.0048, Fig. 4C, D). Taken together, our results showed that in unstable drug-resistant H3122 AR cells, changes in NF-κB phosphorylation after drug rechallenge and withdrawal could be a factor leading to CCN1 alteration, which in turn affected pAKT and VEGFA. Inhibition of NF-κB expression may improve the sensitivity to alectinib in H3122 AR cells.

## Conclusion

ALK rearrangements define a distinct molecular subtype of NSCLC with a favorable clinical response to ALK TKIs. Although ALK inhibitors have produced remarkable outcomes in advanced NSCLC harboring ALK rearrangement, acquired resistance is inevitable, leading to disease progression. Several mechanisms of resistance to ALK-targeted therapy have been identified, including kinase domain mutations, amplifications, and ALK-independent pathway activation [8–10]. Despite the development and application of DNA sequencing in clinical practice, the mechanisms of resistance remain unknown in a substantial proportion of patients, particularly those with non-genomic resistant mutations [35, 36]. It is crucial to investigate the resistance mechanisms in patients in order to rationally select subsequent therapies. Previous studies have reported the occurrence of “reversible resistance,” in which patients resistant to an ALK inhibitor could regain the sensitivity to the same agent after a period of treatment discontinuation. The reversible state of resistance suggests the absence of genetic aberrations. Little is known about the potential mechanisms, so further studies are needed to improve clinical management.

In this study, alectinib was used to construct drug-resistant EML4-ALK mutant H3122 cells and confirmed the existence of reversible drug resistance, i.e., high concentrations of alectinib could maintain the drug-resistant state of the cells, and the cells became sensitive to alectinib after drug removal. In the absence of genetic alterations causing resistance, RNA sequencing was performed to explore changes in the expression profile. CCN1 changes were found in both RNA and protein upon withdrawal or rechallenge with alectinib [27–29]. CCN1 is a member of the CCN family of growth factors, which includes CTGF, NOV, WISP-1, WISP-2, and WISP-3. CCN1 plays an essential role in cell proliferation, survival, adhesion, and angiogenesis [30–32]. High expression of CCN1 has been detected in various cancers such as breast cancer, gastric cancer, and ovarian cancer. Previous studies have indicated that CCN1 can mediate resistance to antitumor therapies in various cancers through diverse mechanisms. CCN1 can activate the RAS-MAPK and PI3K-AKT signaling pathways by stimulating integrin αvβ3 or upregulate Bcl-xL and cIAP1 to cause drug resistance [37, 38]. In breast cancer, the TAZ-TEAD-CCN1/CCN2 signaling axis plays a crucial role in taxol resistance [39]. CCN1 can transcriptionally downregulate ERα expression, leading to tamoxifen resistance [40]. The response to imatinib in chronic myeloid leukemia can be regulated by CCN1 through the NF-κB/Bcl-2 pathway [41]. Furthermore, overexpression of CCN1 can inhibit carboplatin-induced apoptosis by decreasing Bax expression and increasing Bcl-xL, Mcl-1, and Bcl-2 levels in ovarian cancer cells [42]. Despite accumulating studies conducted, the function of CCN1 in lung cancer was not fully understood and remains controversial. Thus, the role of CCN1 in lung cancer remains unclear and deserves further investigation.

In our studies, we found that inhibition of CCN1 could also significantly decrease the proliferation and angiogenesis capacity of H3122 AR cells. CCN1 has been reported to affect tumor growth through its potent angiogenic activity. Our study found that knockdown of CCN1 could cause the downregulation of VEGFA via the AKT pathway, which could lead to the suppression of tumor growth and angiogenesis. In addition, inhibition of CCN1 could render reversibly drug-resistant cells more sensitive to alectinib. Although improved sensitivity to alectinib was observed by inhibiting CCN1, overexpression of CCN1 did not confer resistance to alectinib, indicating that the potential resistance mechanisms in H3122 AR cells are complicated. However, the improved sensitivity to alectinib by inhibition of CCN1 expression or receptor still suggests that CCN1 could serve as a therapeutic target. Further experiments revealed that activated NF-κB could induce CCN1 upregulation, thereby affecting AKT phosphorylation and VEGFA expression.

Although our study demonstrated that sensitivity to alectinib could be restored by inhibiting CCN1, the development of specific targeting agents for CCN1 remains relatively unexplored. Given that CCN1 can interact with integrins such as αvβ3 to mediate diverse functions, blocking these interactions with integrin inhibitors could be a viable strategy. Furthermore, since CCN1 is a secreted protein, the development of novel monoclonal antibodies specifically targeting CCN1 holds significant promise [29, 31].

CCN1 is an immediate-early gene that can be rapidly transcriptionally activated in response to various stimuli [31]. As CCN1 encodes a secreted matricellular protein whose aberrant expression can be detected through liquid biopsies such as serum or saliva samples, recent studies suggest that CCN1 may be useful as a biomarker or therapeutic target in certain diseases. The potential of CCN1 as a biomarker is currently being investigated in diseases such as juvenile idiopathic arthritis (NCT05534347) and acute kidney injury (NCT05242705). Zhang et al. reported that salivary CCN1 mRNA was upregulated in lung cancer compared to matched controls [43]. A pilot study also found that plasma CCN1 levels were significantly elevated in lung cancer patients compared with healthy controls. In our study, the changes in resistance status associated with CCN1 expression may indicate the value of CCN1 as a resistance biomarker [44]. Further research using clinical samples is needed.

Our study had several limitations. In this study, only one ALK TKI was employed to explore the unstable resistant mechanism. Further research is demanded to investigate additional targeted inhibitors, such as lorlatinib. The reversible resistant state was only determined in H3122 AR, and more cell models are needed to confirm the CCN1 function in unstable resistance. In addition, the rapid change of resistant status after alectinib withdrawal makes the animal experiment difficult.

In conclusion, our study firstly demonstrated that CCN1 plays an important role in regulating the reversible resistant state of lung cancer cells. Suppression NF-κB or CCN1 receptor could improve the sensitivity to alectinib, suggesting promising combination targets that deserve further investigation.

## Supplementary information

Supplementary Legends

Figure S1

Figure S2

Figure S3

Figure S4

Figure S5

Figure S6

Figure S7

Table S1

Table S2

Table S3

Western blot raw data

## Data Availability

The datasets generated during and/or analyzed during the current study are available from the corresponding author on reasonable request. The raw sequence data reported in this paper have been deposited in the Genome Sequence Archive [16] in National Genomics Data Center [17], China National Center for Bioinformation/Beijing Institute of Genomics, Chinese Academy of Sciences (GSA-Human: HAR009333) which are publicly accessible at https://ngdc.cncb/ac.cn/gsa-human.
