# Supplementary material for: CCN1 is a therapeutic target upregulated in EML4-ALK mutant lung adenocarcinoma reversibly resistant to alectinib
Source: Cell Death Dis. 2025 Apr 15;16(1):303. doi: 10.1038/s41419-025-07601-4 (PMC12000322; doi:10.1038/s41419-025-07601-4)
Supplement: Supplementary file 1 — Supplementary Legends [file 41419_2025_7601_MOESM1_ESM.docx]

**Supplementary Legends**

**Figure S1. A.** The frequency of emerged mutations detected by WGS in H3122 AR. **B.** The distribution of nonsynonymous mutations (1 In-frame insertion, 6 nonsense mutation and 55 missense mutations). WGS, whole-genome sequencing.

**Figure S2. A.** CCN1 and CCN2 RNA level of samples collected at different time during alectinib holiday-administration-holiday period. **B.** CCN2 protein level detection at different time during alectinib holiday-administration-holiday period.

**Figure S3.** Scratch wound healing assays of H3122 AR after CCN1 knockdown. CCN1 knockdown did not affect the migration ability of H3122 AR. Error bars reflect mean ± standard deviation.

**Figure S4.** KEGG analysis for samples collected at different time during alectinib holiday-administration-holiday period (Cluster 9).

**Figure S5.** Western blotting of H3122 AR treated with SB273005, an integrin receptor.

**Figure S6.** CCN1 transfection did not affect the sensitivity to alectinib in H3122 AR cells.

**Figure S7.** Western blotting of H3122 treated with exogenous CCN1.

**Table S1.** Emerged mutations detected through WGS in H3122 AR compared with H3122. WGS, whole-genome sequencing.

**Table S2.** Summary of different variant types.

**Table S3.** Nonsynonymous mutations detected in H3122 AR.
