# Supplementary figures and images for: CCN1 is a therapeutic target upregulated in EML4-ALK mutant lung adenocarcinoma reversibly resistant to alectinib
Source: Cell Death Dis. 2025 Apr 15;16(1):303. doi: 10.1038/s41419-025-07601-4 (PMC12000322; doi:10.1038/s41419-025-07601-4)

A

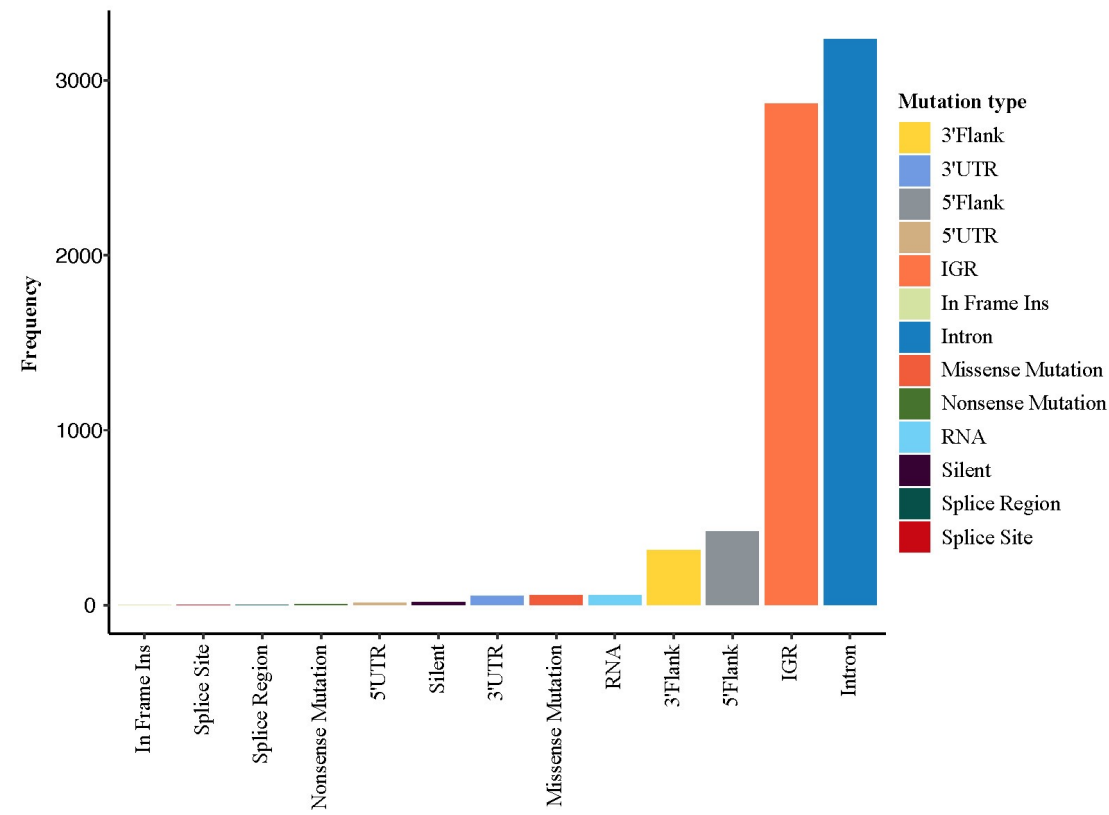

B

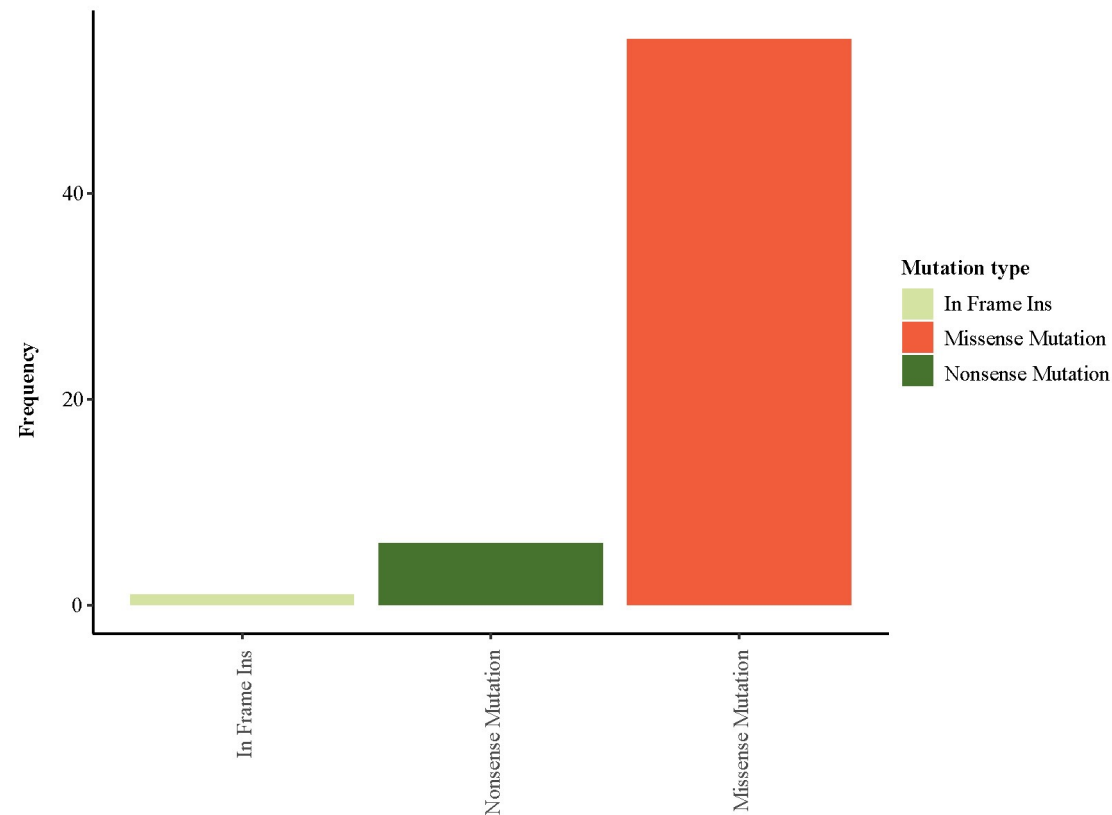

Supplement: Supplementary file 2 — Figure S1 [file 41419_2025_7601_MOESM2_ESM.pdf]

**A**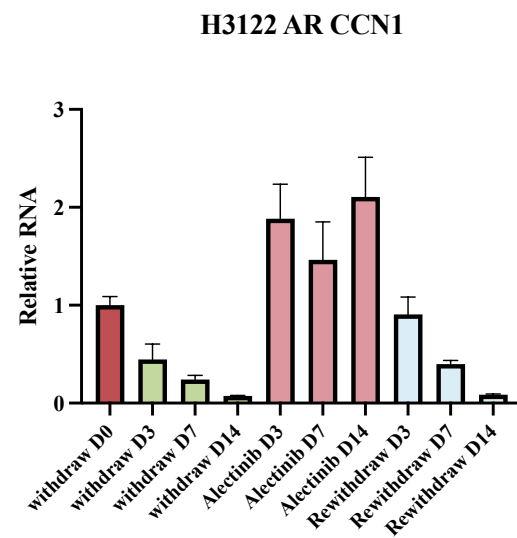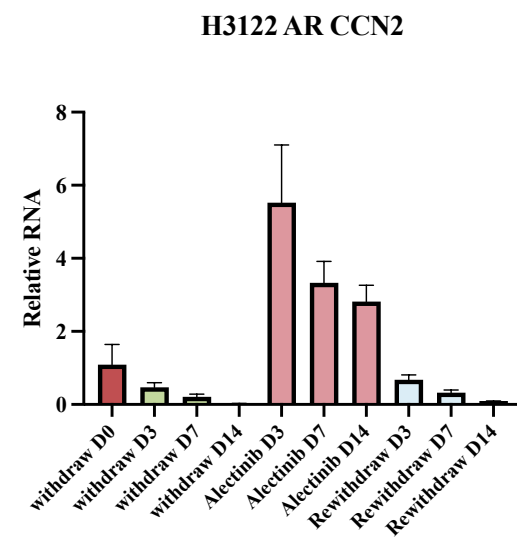**B**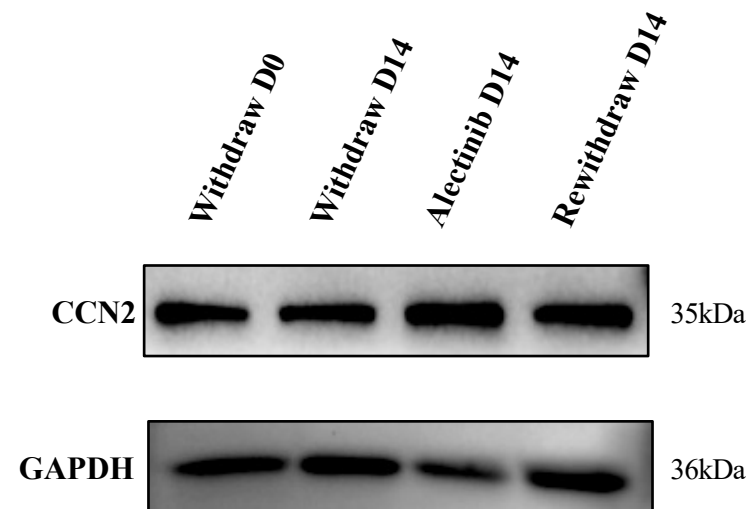

Supplement: Supplementary file 3 — Figure S2 [file 41419_2025_7601_MOESM3_ESM.pdf]

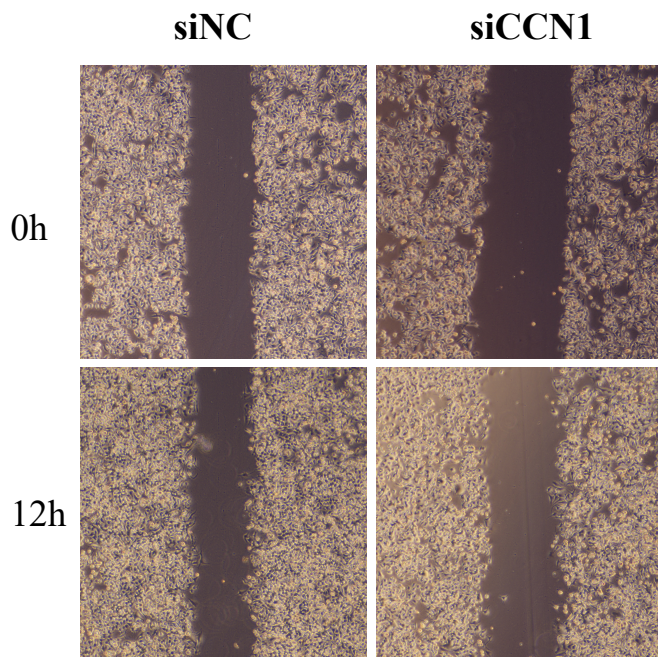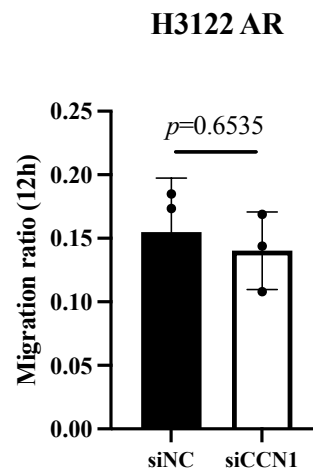

Supplement: Supplementary file 4 — Figure S3 [file 41419_2025_7601_MOESM4_ESM.pdf]

Figure S4

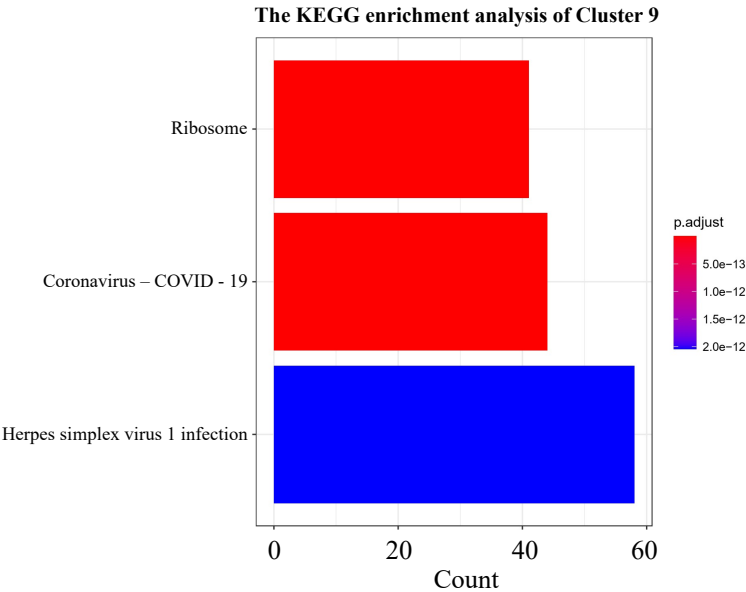

Supplement: Supplementary file 5 — Figure S4 [file 41419_2025_7601_MOESM5_ESM.pdf]

### H3122 AR

SB273005 1UM

-

8h

CCN1

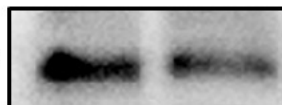

41kDa

AKT

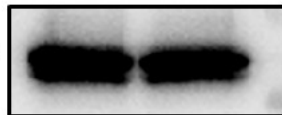

60kDa

pAKT

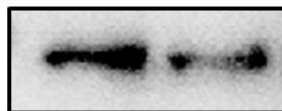

60kDa

VEGFA

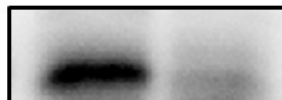

23kDa

GAPDH

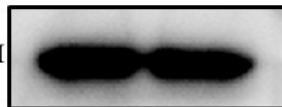

36kDa

Supplement: Supplementary file 6 — Figure S5 [file 41419_2025_7601_MOESM6_ESM.pdf]

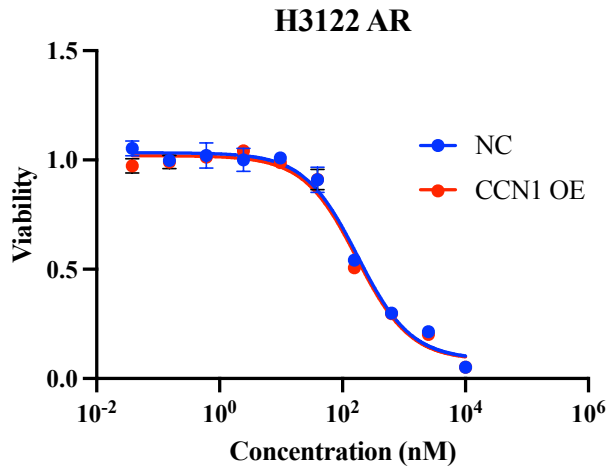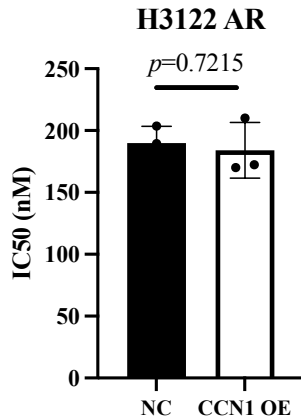

Supplement: Supplementary file 7 — Figure S6 [file 41419_2025_7601_MOESM7_ESM.pdf]

## H3122

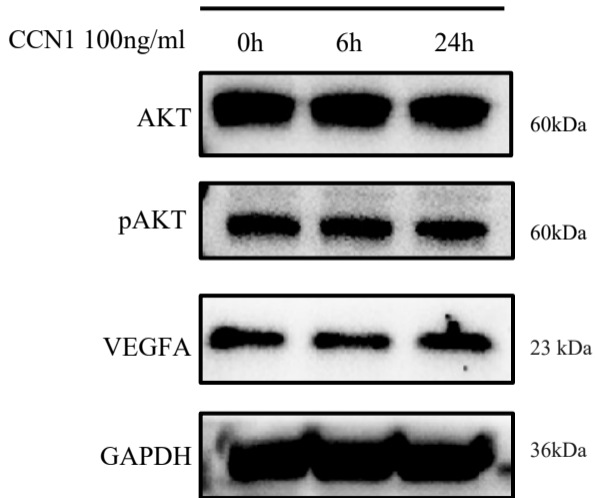

Supplement: Supplementary file 8 — Figure S7 [file 41419_2025_7601_MOESM8_ESM.pdf]
