# Supplementary material for: CCN1 is a therapeutic target upregulated in EML4-ALK mutant lung adenocarcinoma reversibly resistant to alectinib
Source: Cell Death Dis. 2025 Apr 15;16(1):303. doi: 10.1038/s41419-025-07601-4 (PMC12000322; doi:10.1038/s41419-025-07601-4)
Supplement: Supplementary file 10 — Table S2 [file 41419_2025_7601_MOESM10_ESM.docx]

**Table S2. Summary of different variant types.**

| **Variant types** | **Number** |
| --- | --- |
| Intron | 3237 |
| IGR | 2868 |
| 5’Flank | 420 |
| 3’Flank | 313 |
| Missense mutation | 55 |
| RNA | 55 |
| 3’UTR | 51 |
| Silent | 19 |
| 5’UTR | 12 |
| Nonsense mutation | 6 |
| Splice region | 3 |
| Splice site | 1 |
| In_frame insertion | 1 |

IGR, Inverted Gene Rearrangement; UTR, untranslated region.
