# Supplementary material for: CCN1 is a therapeutic target upregulated in EML4-ALK mutant lung adenocarcinoma reversibly resistant to alectinib
Source: Cell Death Dis. 2025 Apr 15;16(1):303. doi: 10.1038/s41419-025-07601-4 (PMC12000322; doi:10.1038/s41419-025-07601-4)
Supplement: Supplementary file 12 — Western blot raw data [file 41419_2025_7601_MOESM12_ESM.docx]

**Figure 1F**


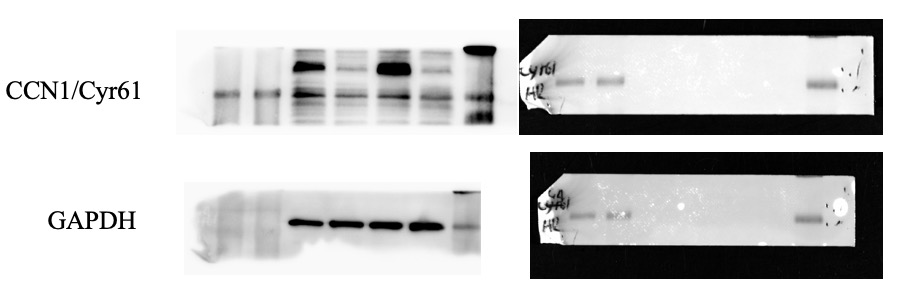


**Figure 3C**

The proteins in the blue box were the proteins shown in the manuscript, and the first column was the H3122 protein.

**
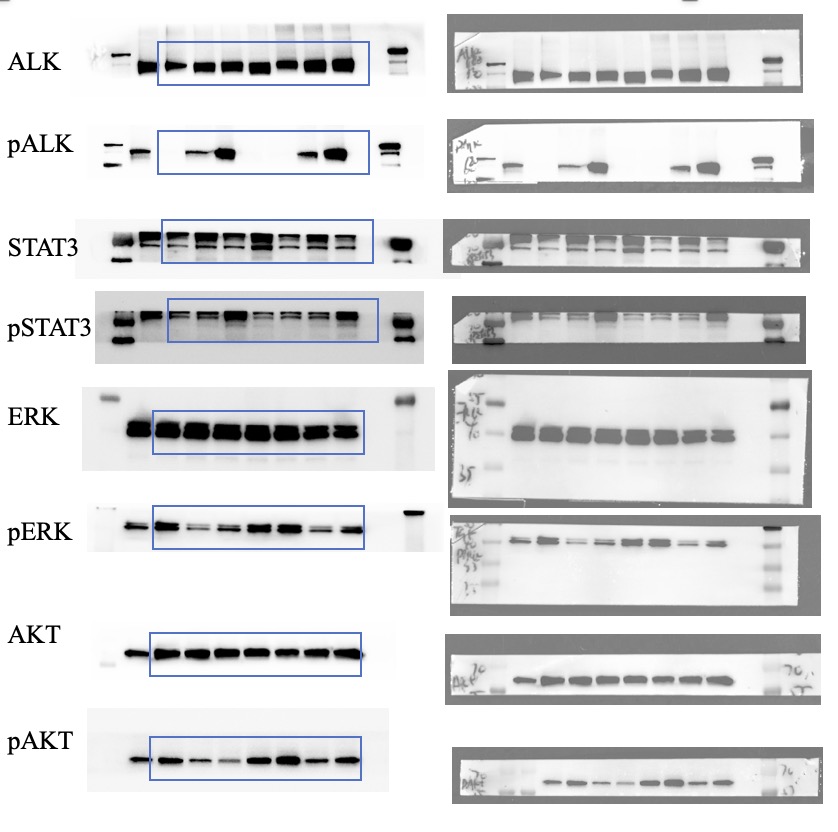
**

**
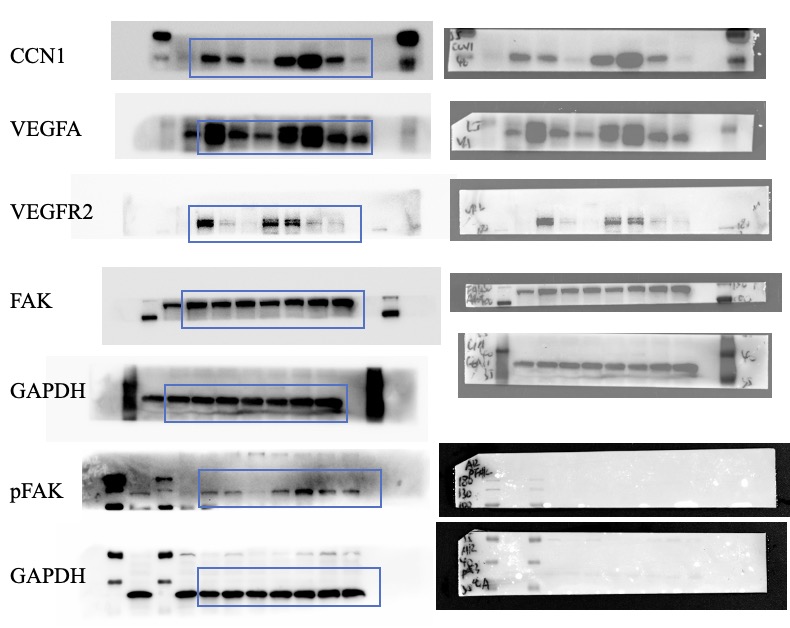
**

**Figure 3D**

**
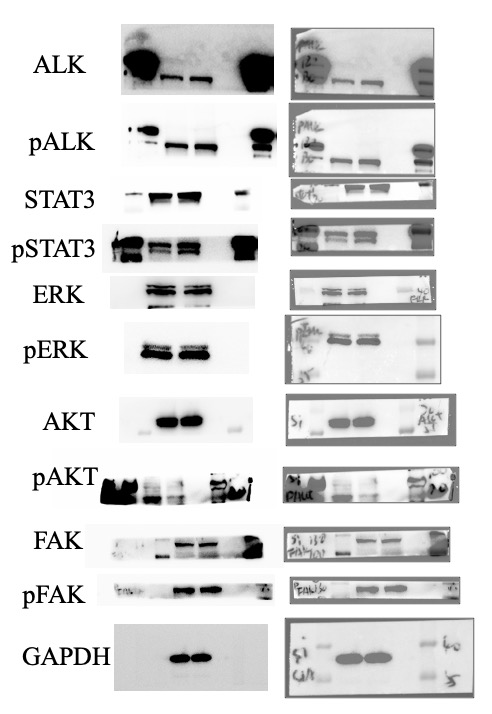
**

**
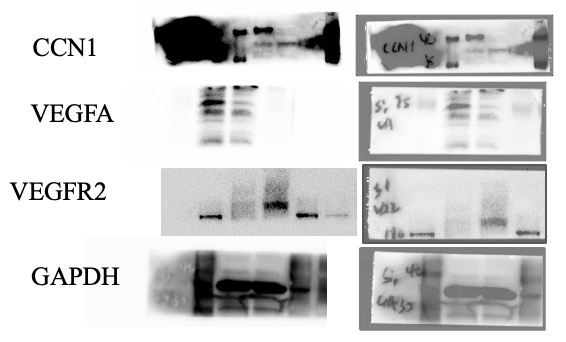
**

**Figure 3E**

**
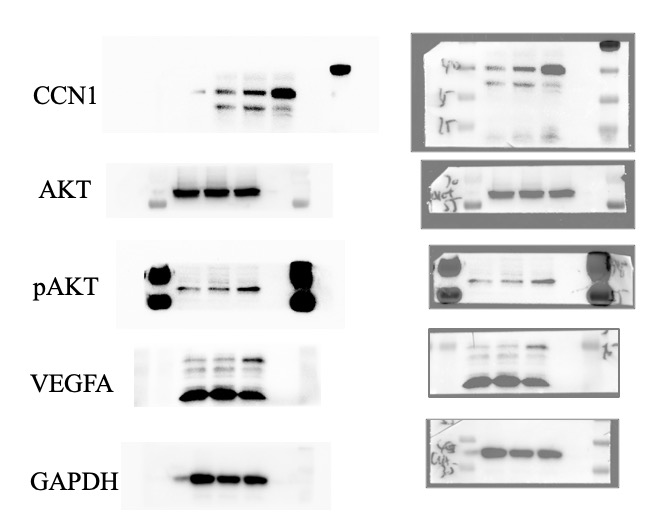
**

**
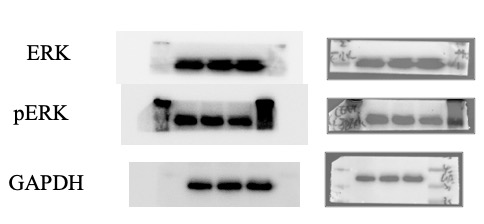
**

**Figure 3F**

**
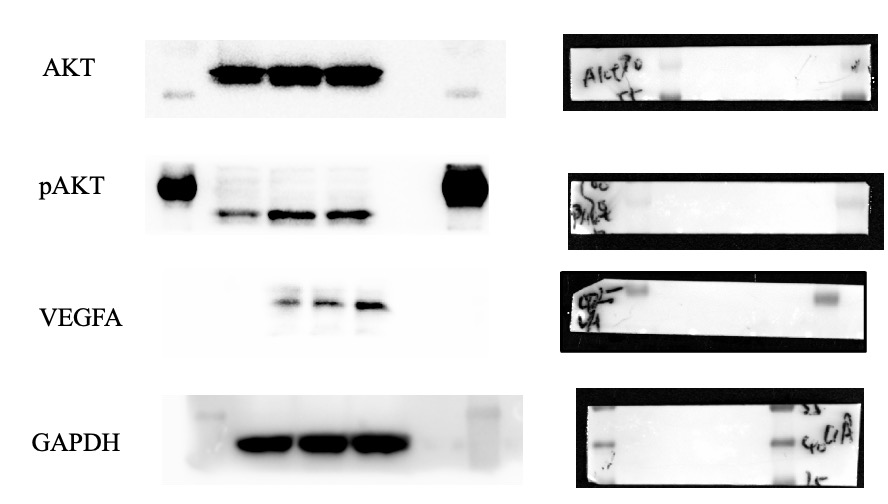
**

**Figure 3G**

**
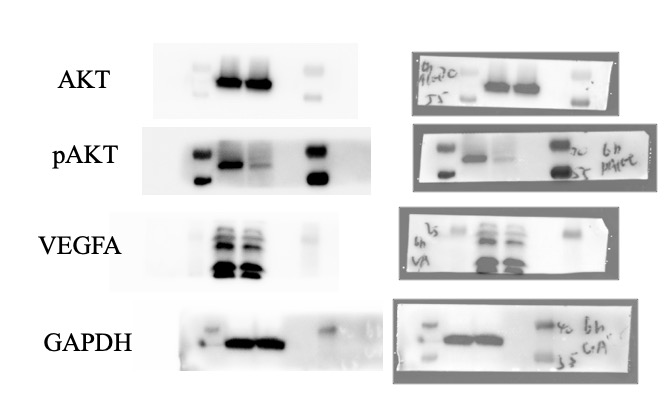
**

**Figure 4A**

The proteins in the blue box were the proteins shown in the manuscript. And the first column was the H3122 protein.

**
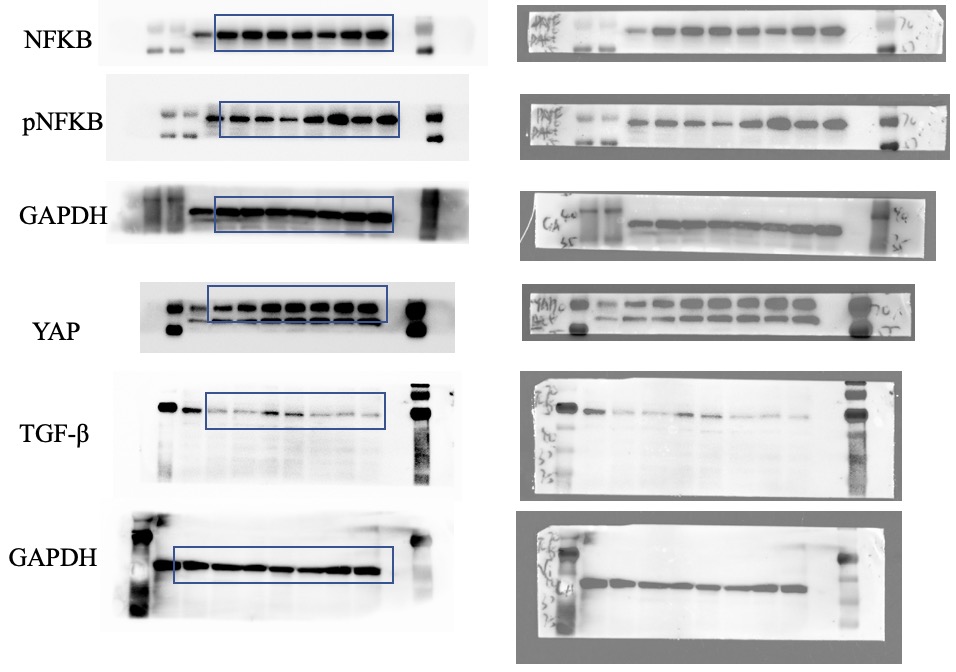
**

**Figure 4B**

**
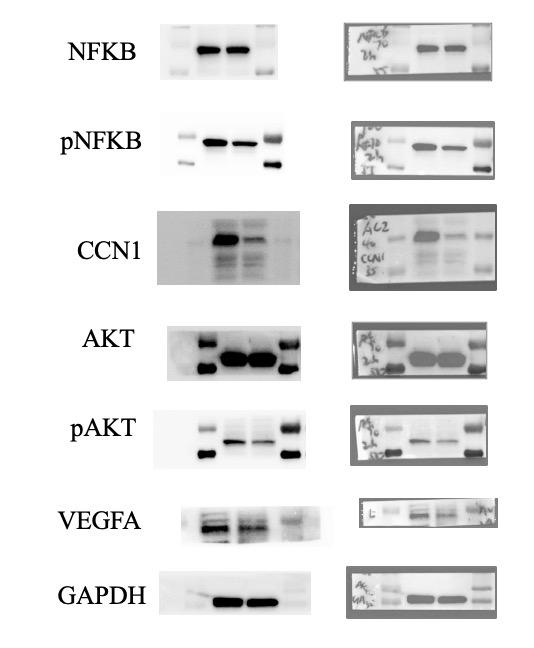
**

**Figure S2B**

**
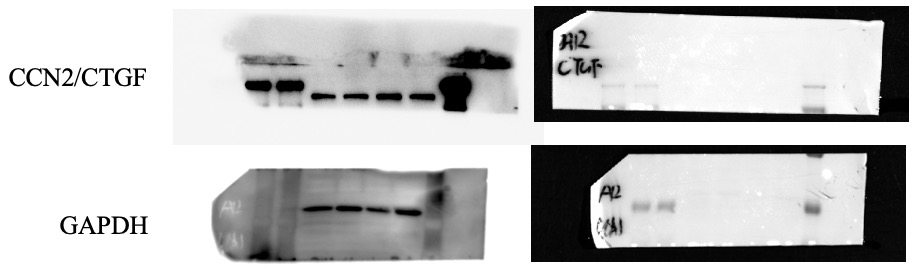
**

**Figure S5**

**
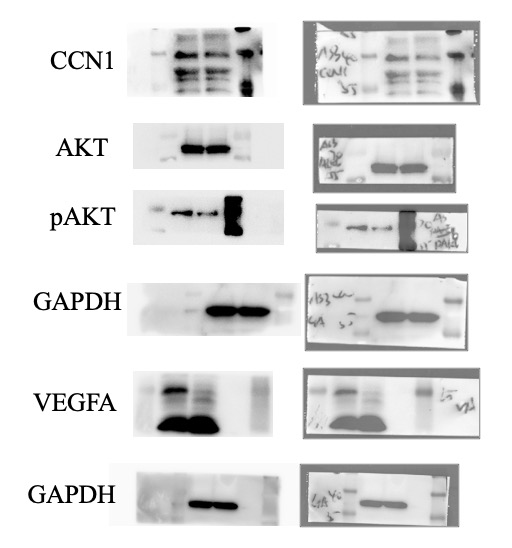
**

**Figure S7**

The proteins in the blue box were the proteins shown in the manuscript. The other proteins came from repeated experiment.

**
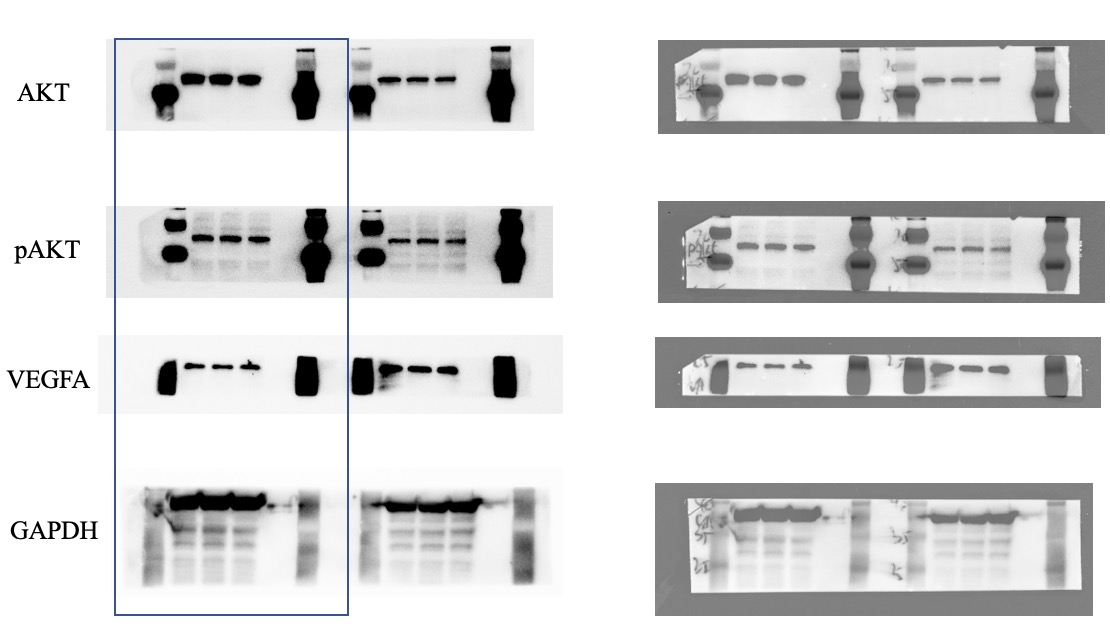
**
